# Association between Pesticides in House Dust and Residential Proximity to Farmland in a Rural Region of Taiwan

**DOI:** 10.3390/toxics9080180

**Published:** 2021-07-30

**Authors:** Sailent Rizki Sari Simaremare, Chien-Che Hung, Tzu-Hsien Yu, Chia-Jung Hsieh, Lih-Ming Yiin

**Affiliations:** 1 Institute of Medical Sciences, Tzu Chi University, 701, Sec. 3, Zhongyang Road, Hualien City 970374, Taiwan; 103324121@gms.tcu.edu.tw; 2 Department of Public Health, Tzu Chi University, 701, Sec. 3, Zhongyang Road, Hualien City 970374, Taiwan; gavink23@gms.tcu.edu.tw (C.-C.H.); tzuhsien0119@gms.tcu.edu.tw (T.-H.Y.); cjhsieh@mail.tcu.edu.tw (C.-J.H.); 3 TCU Center for Health and Welfare Data Science, 701, Sec. 3, Zhongyang Road, Hualien City 970374, Taiwan

**Keywords:** agriculture, geographic information system, house dust, pesticides, Taiwan

## Abstract

Pesticide drift was reported in many international studies, but rarely studied in Taiwan. We conducted a study in a rural region of Taiwan to examine the associations between pesticides in house dust and nearby agricultural areas using geographic information system (GIS). A questionnaire regarding home characteristics and pesticide use, and indoor and outdoor dust samples were collected from 47 rural homes. Dust samples were analyzed for six pesticides, and agricultural land data for GIS analysis were retrieved from a national website. All but prallethrin were frequently detected from indoor dust samples (>50%), and the maximum concentrations were all below 1000 ng/g. Detection frequencies and concentrations of pesticides in outdoor dust samples were even lower than that in indoor dust samples. Only “work involving pesticides” in the questionnaire was significantly associated with four pesticides in house dust (*p* < 0.05). Carbofuran and tetramethrin in house dust were significantly correlated with rice cultivation area at certain buffer distances (*ρ* > 0.33, *p* < 0.05), and chlorpyrifos was found to be associated with abandoned cultivation area, suggesting the occurrence of pesticide drift. Despite the low levels of pesticides in house dust, residents in the rural region should be cautious of pesticide drift from nearby active or abandoned farmlands.

## 1. Introduction

House dust as an exposure source of chemical contaminants has been gaining attention over the years [1]. Applications of pesticides inside and/or outside the households on a regular basis could result in pesticide accumulation in dust through everyday human and/or animal activities. Without frequent cleaning, the pesticide-contaminated dust could serve as a source of combined exposure via dietary and non-dietary routes [2]. Many pesticide-related studies have been conducted to assess pesticide residue in house dust, and most of them have further indicated the correlation between the pesticide concentrations found in indoor dust and agricultural areas in the proximity [3,4,5,6,7,8,9,10]. For example, a field study conducted in California, USA, indicated that low-income children were “potentially exposed to a mixture of pesticides as a result of poorer housing quality,” which included contamination from agricultural use of pesticides in the proximity [4]. The indicated relation suggests the occurrence of pesticide drift, which reasonably leads to an association of pesticides between indoor and outdoor environments. A Dutch study indicated a moderate relation between indoor and outdoor concentrations of airborne pesticides [11]; additionally, a Korean study confirmed that pesticide residue in indoor dust resulted from the infiltration of pesticide particles from outdoors [12]. Our previous pilot study, however, did not indicate any relation between indoor and outdoor pesticide concentrations, likely due to different sources and/or environmental fates [3].

Recently, geographic information system (GIS) has been considered to be a valuable tool to monitor, analyze and model pesticide migration in the environment and the related health impacts [13]. It was vastly used in pesticide studies to assess residential exposures and their correlation with some diseases [14,15,16], or to correlate residential exposures to nearby agriculture areas in determining a drift pathway [3,17,18,19,20]. Although GIS has been widely applied to pesticide studies internationally, it is rarely used for studying pesticide exposure in Taiwan. Our previous study using GIS as approach found that chlorpyrifos and cypermethrin were the most detected pesticides in indoor and outdoor dust of a rural region in Taiwan, and that chlorpyrifos in outdoor dust was associated with farms in the proximity of the homes [3]. Despite the inspiring association identified by the pilot study, there were questions remaining to be answered; besides, many houses sampled in the study were located in areas with urban or suburban settings, which might have attenuated the study effect. Thus, we developed a new study attempting to further explore pesticide exposure, and to confirm the possible pesticide drift in the countryside of Taiwan.

In this study, we focused on dust sampling from houses that were truly located in the countryside to avoid any interference caused by urban or suburban settings. We analyzed four major groups of pesticides from the samples, including carbamate, organophosphate, phenylpyrazole, and pyrethroid, and used GIS to assess potential pesticide drift from agricultural areas to residential units nearby. This study herein not only confirmed the result obtained from the pilot study, but also provided more information regarding pesticide exposure in a rural region of Taiwan.

## 2. Materials and Methods

### 2.1. Participating Houses in the Study and Sample Collection

**Figure 1:**
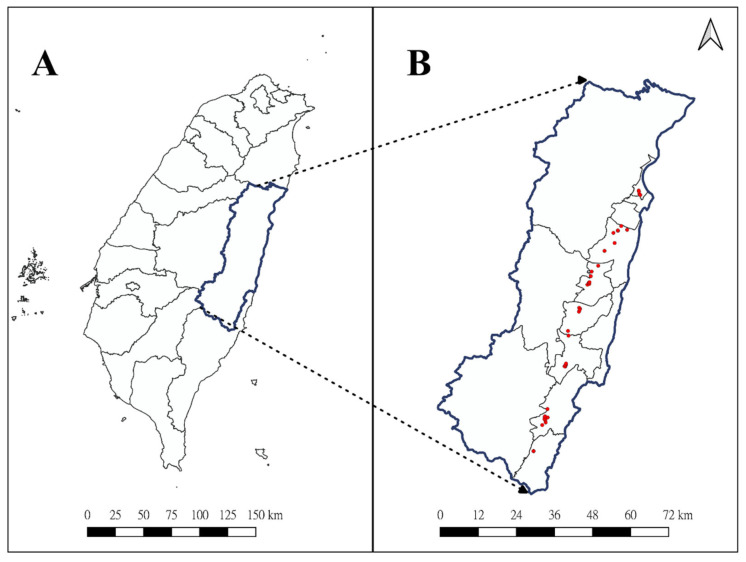
Location of Hualien County in Taiwan (**A**) and that of sampling houses in Hualien County (**B**).

Similar to the previous pilot study [3], this study was also conducted in Hualien county, which was one of the least industrialized areas in Taiwan with active farmlands of 42.7 kha (retrieved from 2019 annual report of the Council of Agriculture, Executive Yuan, R.O.C., https://eng.coa.gov.tw/, accessed on: 7 May 2021). In contrast to the previous study in which participating homes were urban-like, we selected 47 houses located in the countryside along the East Rift Valley, known for scenic views of farmlands and the associated agricultural produce (Figure 1). After obtaining the consent, we used a vacuum sampler to collect a composite sample of indoor dust and a grab sample of outdoor dust in the front door at each household; details of dust sampling were described previously [3]. The questionnaire, which consisted of 13 questions regarding home characteristics and pesticide use, was conducted after the dust sampling. All home visits and data collection were administered during a period between August 2018 and June 2019. The study protocol and questionnaire were reviewed and approved by the Ethical Committee of Tzu Chi General Hospital/University (no.: IRB107-01-B, approved on 1 February 2018).

### 2.2. Sample Treatment and Analysis

We selected the analytes from four major groups of widely used pesticides, because of concern for their detrimental effect on the environment and human health. The pesticides for analysis were carbofuran (carbamate), chlorpyrifos (organophosphate), fipronil (phenylpyrazole), and cypermethrin, prallethrin and tetramethrin (pyrethroids). Of the six analytes, carbofuran was identified as one of the pesticides with the highest concern for human health risks in Taiwan [21]. In addition, carbofuran has been used for agriculture only, and thus its residue in house dust could be considered evidence of pesticide drift. Three internal standards of carbaryl, imiprothrin and parathion-methyl were used in the analysis for quality assurance. The standards of carbofuran (≥98%), chlorpyrifos (≥98%), cypermethrin (≥90%) and tetramethrin (99%) plus three internal standards (~100%) were purchased from Millipore Sigma (St. Louis, MO, USA), whereas fipronil (98%) and prallethrin (95%) were obtained from Toronto Research Chemicals Inc. (North York, ON, Canada).

We modified a reliable analytic method using gas chromatography-mass spectrometry (GC-MS) to analyze the six pesticides simultaneously [3]. In brief, we weighed 0.5 g of dust from each sample, and added 10 mL ethyl acetate for 30 min ultrasonic extraction. The sample solution was concentrated, reconstituted with 400 µL ethyl acetate, filtered, and transferred to an insert vial for GC-MS analysis (7890/5975 Agilent Technologies, Santa Clara, CA, USA).

The GC-MS instrument was equipped with a capillary column (HP-5MS column, 30 m × 0.25 mm × 0.25 µm, Agilent Technologies, Santa Clara, CA, USA). We used ultra-purity helium (99.9995%) as carrier gas, and set a constant flow rate at 0.7 mL/min; the injection volume was 1 μL, and the inlet condition was set at 280 °C with a split model (10:1). A pre- and post-washing and baking program was administered to avoid cross- contamination and contaminants with high boiling points. The temperature was set at 100 °C for one minute in the beginning, increased to 260 °C at a rate of 20 °C/min, increased to 280 °C at 10 °C/min, and held at 280 °C for one minute (total runtime: 12 min). The post-run lasted 1 min at 300 °C. We used selective ion monitoring mode to enhance the sensitivity of detection; the limits of detection were determined to be 0.17 ng/g or lower for the six pesticides. The recovery rates were determined to be 93% or above by spiking samples with standards; the coefficients of variance of the overall analysis were lower than 6%.

### 2.3. Data Management and Statistical Analysis

To find out a possible seasonal effect, dust samples collected within the period between November 2018 and April 2019 were categorized into a cold season group, and samples taken in the other months were labeled as warm season samples. Pesticide concentrations were log-transformed for normalization prior to linear regression analysis, which was performed to relate the pesticide contents in dust with the questionnaire responses. The derived β in the regression analysis was converted to the percent change of pesticide concentration corresponding to a factor of questionnaire response using Formula 1, because the dependent variable of pesticide concentration was log-transformed.

(1) $$\%\;\mathrm{change}\;\mathrm{in}\;\mathrm{pesticide}\;\mathrm{concentration}=(e^{\beta }-1)\times 100$$

We conducted univariate linear regression analysis first to find out significant questionnaire factors that were possibly associated with pesticide concentrations in dust, and multivariate regression analysis with those significant factors to confirm their significance. Pesticides with detection frequencies under 50% were excluded from regression analysis, because of lack of representativeness. Spearman correlation analysis was used to examine the associations among pesticide concentrations in indoor and outdoor dust, and between residential pesticide concentrations and the percentages of agricultural areas within buffer distances of 50 m, 150 m, and 250 m. Halves of LODs were used for those non-detects in the analysis [22]. Descriptive statistics was computed in Microsoft^®^ Excel, and other statistical analyses were performed using SPSS statistical software package version 23.0 (SPSS Inc., Chicago, IL, USA, 2015). 

### 2.4. Geographic Information System (GIS)

The application of GIS for assessing pesticide exposure was similar to that described previously [3]. The location datum in TWD97 Coordinate System of each participating house was determined by a handheld global positioning system navigator (GPSMAP 60CSx, Garmin^®^, New Taipei City, Taiwan) and checked with Taiwan National Land Surveying and Mapping Center Service (https://maps.nlsc.gov.tw/, accessed on: 3 August 2020). The location data were used in ArcGIS software (Version 10.6.1, Esri, Redlands, CA, USA, 2018) that was mapping with land use data of agriculture to calculate the percentages of surrounding farmland areas. In this study, categories of non-specific agriculture land (0101), rice cultivation land (010101), crops’ cultivation land (010102), fruit cultivation land (010103), and abandoned agriculture land (010104) were applied in the GIS analysis; the buffer zones for analysis were those regions surrounding each house within radii of 50 m, 150 m, and 250 m.

## 3. Results

Among the six pesticides, tetramethrin was the most frequently detected one (78.7%) from indoor dust samples, followed by cypermethrin (72.3%) and chlorpyrifos (68.1%) with mean concentrations of 83.07 ng/g, 60.25 ng/g, and 53.81 ng/g, respectively (Table 1). As for outdoor dust samples, fipronil (48.9%) was the most frequently detected pesticide, followed by chlorpyrifos (36.2%) and tetramethrin (34.0%) with mean concentrations of 25.01 ng/g, 41.15 ng/g, and 38.19 ng/g, respectively. Of both indoor and outdoor dust samples, fipronil was prevalently detected (~50%), whereas the other pesticides (e.g., carbofuran, tetramethrin) were detected much more frequently in indoor dust than outdoor dust. Overall, the detection frequencies were higher for indoor samples than outdoor counterparts, and so were the concentrations except that of prallethrin.

The questionnaire data and its associations with pesticide concentrations are shown in Table 2. Prallethrin in indoor dust and all in outdoor dust are excluded due to the low detection frequencies (<50%). Most of the questionnaire responses were not significantly associated with pesticides except “work involving pesticides” (9 out of 47 responses, 9/47), which resulted in statistical significance with four pesticides by univariate linear regression analysis (*p* < 0.05). Besides, fipronil in indoor dust was significantly related to “pets at home” (23/47) and “tick treatment for pets” (20/47), whereas cypermethrin was linked with “indoor insecticide application” (27/47). Multivariate regression analysis was performed to find out the true significance of these multiple factors associated with fipronil or cypermethrin. The result indicated that only “work involving pesticides” remained significant to be linked with fipronil or cypermethrin in indoor dust (*p* < 0.05) (Table 3). It is suggested that indoor sources of insecticides (e.g., treatment for pets, domestic application) could be irrelevant to fipronil or cypermethrin in indoor dust.

Spearman correlation analysis indicated that moderate or poor correlations (*ρ* < 0.5) among pesticide concentrations in indoor dust except those between carbofuran and chlorpyrifos (*ρ* = 0.658, *p* < 0.01) and between carbofuran and tetramethrin (*ρ* = 0.529, *p* < 0.01) (data not shown in table). The correlations between the same pesticides in indoor and outdoor dust were mostly strong, including chlorpyrifos (*ρ* = 0.840, *p* < 0.01), fipronil (*ρ* = 0.769, *p* < 0.01), cypermethrin (*ρ* = 0.674, *p* < 0.01), and tetramethrin (*ρ* = 0.690, *p* < 0.01) (Table 4). Carbofuran in indoor dust was not only correlated well with chlorpyrifos and tetramethrin in indoor dust, but also with them in outdoor dust (*ρ* = 0.549 and 0.575, respectively). Although the correlation between carbofuran in indoor and outdoor dust was not as strong as those of the four abovementioned pesticides, it was significant enough to indicate the relation between carbofuran in indoor and outdoor dust (*ρ* = 0.407, *p* < 0.05). 

Table 5 indicated the Spearman correlations between pesticide concentrations in house dust and percentages of agricultural land use within three buffer distances (50 m, 150 m, and 250 m). It appeared that the percentage of agricultural land use increased with the buffer distance, indicating the characteristics of countryside for the sampling houses. None of fipronil, prallethrin and cypermethrin in house dust was significantly correlated with agricultural land use within any buffer zone; thus, those data were excluded from Table 5. Carbofuran in indoor dust was correlated significantly with the non-specific agriculture land and rice cultivation area within all buffer zones (*ρ* = 0.321~0.404, *p* < 0.05), but that in outdoor dust was limited to correlate with rice cultivation area (*ρ* = 0.337~0.365, *p* < 0.05). Chlorpyrifos was significantly associated with abandoned cultivation area within certain buffer zones (*ρ* = 0.311~0.387, *p* < 0.05), whereas tetramethrin in indoor and outdoor dust was found to relate to rice cultivation area at the buffer distance of 150 m (*ρ* = 0.338, 0.420, *p* < 0.05). None of the pesticides was associated with crops’ cultivation area or fruits’ cultivation area.

## 4. Discussion

As expected, the detection frequencies and concentrations of pesticides in indoor dust were higher than that in outdoor dust, probably because of protection from degradation provided by indoor environments and/or existence of indoor sources. The environmental and questionnaire data found one and only significant association between several pesticides (i.e., carbofuran, fipronil, cypermethrin, tetramethrin) and the factor of “work involving pesticides,” suggesting that pesticides could be carried in from the workplace. “Indoor insecticide application” and “pets at home/tick treatment for pets” were considered possible indoor sources of pesticides in dust [4,23,24], but the contribution was shown to be insignificant by multivariate regression analysis. There were only 9 out of 47 homes reporting “yes” to the question of “work involving pesticides,” indicating that the majority did not have specific sources of pesticides found in indoor dust. Thus, the probability that pesticides came from farmlands in the proximity was reasonable.

As shown by other studies [11,12], the association between pesticides in indoor and outdoor environments is confirmed and serves as evidence of pesticide drift. We found strong and significant correlation coefficients for several pesticides’ indoor-outdoor pairs (i.e., chlorpyrifos, fipronil, cypermethrin, tetramethrin), which were in support of the proposed drift pathway (Table 4). The exception of prallethrin reflected the fact that it was not for agricultural use but a common ingredient in the products of mosquito repellent. Besides, the low detection rate of prallethrin in indoor and outdoor dust might be due to its fast degradation in the environment, especially when it was exposed to sunlight [25].

Carbofuran and tetramethrin in indoor or outdoor dust were significantly correlated with the GIS-determined percentages of rice cultivation area, suggesting that pesticide drift from rice farmlands in the proximity could have occurred in the countryside of Hualien. In accordance with the Pesticide Information Query of Taiwan’s Bureau of Animal and Plant Health Inspection and Quarantine (https://pesticide.baphiq.gov.tw/, accessed on: 23 May 2021), both pesticides were known to be applied for rice cultivation in Taiwan, supporting the suggestion. It is interesting to know that tetramethrin is never registered for use in agricultural products but practically used for rice cultivation, unlike carbofuran that is known for an ingredient of common agricultural pesticide products. Another interesting finding is that chlorpyrifos was significantly associated with abandoned cultivation area. It is inferred that chlorpyrifos could be used for control of pests, which massively breed in a suitable environment, such as abandoned lands where wild vegetation grows [26]. In summary, carbofuran, chlorpyrifos and tetramethrin were likely brought into the houses from nearby active or abandoned farmlands; the strong correlations among these three pesticides in indoor and outdoor dust might just partly serve as evidence of pesticide drift.

Compared with our pilot study that was conducted in rural homes with urban settings [3], this study had several similarities and differences. Chlorpyrifos, cypermethrin and tetramethrin were similarly the most frequently detected pesticides in indoor dust, and the detection frequencies were quite close. It is believed that these pesticides were commonly used for domestic or agricultural purposes throughout the county, regardless of the urbanization of townships. The dominance of chlorpyrifos and cypermethrin was in agreement with a Chinese study, which measured airborne pesticides in urban communities of Guangzhou [27]. The seasonality effect shown in that study, however, was not seen in this work, probably because of the limited sample size. A major difference between our previous and current studies was the relatively high detection rates of carbofuran in indoor and outdoor dust (53.2% and 19.1%, respectively) shown by this work, suggesting that houses in the countryside could be subject to exposure to agricultural pesticides. With the results showing carbofuran present in house dust and its significant correlation with nearby cultivation areas, we conclude that pesticide drift from nearby farmland activities could actually occur to houses in the countryside.

The trace of carbofuran in the home environment is of concern. Due to its high toxicity, some countries have banned the use of carbofuran for agriculture [28]. In Taiwan, while products with high percentages of carbofuran (>37.5%) were banned in 2017, this study was able to find trace-level carbofuran in dust inside 25 rural houses (53.2%), suggesting prevalent use of carbofuran-containing products in the area. We were unsure whether the banned products were used, but given the detection result, the possibility of illegal use of the banned products could not be ruled out. Chou et al. reported the concern of high risk of carbofuran and compelled the need for monitoring of carbofuran use in Taiwan [21]. Based on the finding regarding carbofuran in house dust, we suggest that the proposal made by Chou et al. should be implemented for lowering the risk of carbofuran to farmers and their neighbors.

Despite the similar detection frequencies of chlorpyrifos, cypermethrin and tetramethrin in indoor dust, the concentrations detected in this study were apparently lower than that of the previous pilot study [3]. The major reason for this difference in concentration was probably due to indoor sources of pesticides. In this study, we did not find any significant association between pesticide concentrations in dust and indoor sources (e.g., indoor insecticide application), and the pesticides in indoor and outdoor dust were correlated well, suggesting that sources of pesticides could primarily come from nearby farmlands. In contrast, the indoor-outdoor correlations of the same pesticides were poor in the previous pilot study, indicating different sources for the indoor and outdoor environments. Most of the homes in that study were in urban or suburban areas, where indoor insecticide application was likely administered. Thus, the indoor concentrations found in that study were reasonably higher than that in this one. 

Compared with data from other pesticide studies, the concentrations found in this study were also relatively low. For example, a Californian study reported a median concentration of trans-permethrin in indoor dust to be 504 ng/g [4], whereas our study had a median value of 17.96 ng/g for cypermethrin. A median concentration of chlorpyrifos was reported to be 70 ng/g for the least influenced homes of Central Washington State, USA [8], which was higher than our median concentration of chlorpyrifos in indoor dust, 14.58 ng/g. In a French study, permethrin was the most abundant pesticide with a median concentration of 770 ng/g [29], which was more than 40 times higher than that of cypermethrin (17.96 ng/g) found in our study. The relatively low levels of pesticides in house dust of a rural region in Taiwan could be explained by two reasons, the way of pesticide application and the climate. Unlike the USA where mechanical pesticide applications are feasible, almost all pesticide applications in Taiwan are handled manually; by doing so, the distribution of pesticide beyond the cropland should be limited, compared to that by mechanical applications in the USA. Thus, a limited pesticide drift in Taiwan could lead to low concentrations of pesticides in house dust. As for the climate, Taiwan is known for an island receiving abundant precipitation yearly; according to the precipitation data derived from Taiwan’s Central Weather Bureau (https://www.cwb.gov.tw/V8/E/D/DailyPrecipitation.html, accessed on: 6 June 2021), the average rainy days in a month was about 15 days with mean monthly precipitation of 138 mm during the study period. It can be imagined that frequent rain could wash away certain levels of pesticides in the environment, and subsequently result in low concentrations in house dust. Additionally, organic farming, which is evidenced to promote pest control as well as to reduce use of pesticides [30], has been promoted in the area by Hualien District Agricultural Research and Extension Station for decades [31]. The low levels of pesticides in house dust as presented in this study could serve as a result from the effort of organic agriculture.

This study has several limitations. First of all, because GIS is based on estimation of agricultural surface areas at a specific distance from a sampling location, land use or type of cultivation that is not up to date might affect the accuracy of the outcome. Secondly, we could not obtain the information of pesticides used in Hualien, but selected several commonly used ones in the study; fortunately, we were able to find out associations in support of pesticide drift pathway. Finally, our small sample size might limit the statistical power and lead to insignificant results for regression analysis; however, we still gained several significant data, which were confirmed to agree with the study hypothesis. Despite the limitations, our study has a strength that is worth mentioning. The use of GC-MS that was able to detect trace levels of pesticides in dust helped gain the data and subsequently the result of the study. Had we not used GC-MS, the low levels of pesticides in house dust could have become a number of non-detects, which led to insignificant associations and no support for pesticide drift.

## 5. Conclusions

Chlorpyrifos, cypermethrin and tetramethrin were the most frequently detected pesticides in indoor dust of a rural region in Taiwan. Except “work involving pesticides” in the questionnaire was associated with pesticide concentrations in indoor dust, no significant indoor sources of pesticides were observed, indicating that pesticides indoors could primarily originate from outdoors. Carbofuran, chlorpyrifos and tetramethrin in house dust were significantly associated with nearby agricultural area percentages (i.e., rice cultivation area, abandoned cultivation area) determined by GIS, suggesting the occurrence of pesticide drift. Despite the low levels of pesticides present in the indoor environment, people living in the rural region should be cautious of the probability of pesticide drift from active farmlands or abandoned cultivation areas in the proximity.

## Figures and Tables

**Table 1:** Pesticide concentrations of indoor and outdoor dust (*n* = 47).

Sample Type	Pesticide	DF (%)	Mean ± SEM (ng/g)	50th Percentile (ng/g)	75th Percentile (ng/g)	Maximum (ng/g)
Indoor	Carbofuran	53.2	8.95 ± 1.99	4.33	13.32	74.30
	Chlorpyrifos	68.1	53.81 ± 11.05	14.58	88.94	398.31
	Fipronil	66.0	34.31 ± 5.91	19.25	50.52	143.36
	Prallethrin	34.0	4.37 ± 1.26	<LOD	8.25	50.50
	Cypermethrin	72.3	60.25 ± 12.05	17.96	104.25	388.65
	Tetramethrin	78.7	83.07 ± 13.67	47.33	114.54	313.16
Outdoor	Carbofuran	19.1	7.31 ± 3.36	<LOD	<LOD	117.80
	Chlorpyrifos	36.2	41.15 ± 14.10	<LOD	21.57	471.25
	Fipronil	48.9	25.01 ± 6.18	<LOD	33.84	193.60
	Prallethrin	6.4	5.70 ± 3.28	<LOD	<LOD	113.25
	Cypermethrin	34.0	38.19 ± 15.04	<LOD	14.30	507.52
	Tetramethrin	25.5	39.03 ± 15.37	<LOD	13.36	608.37

DF: detection frequency; SEM: standard error of mean; <LOD: under limit of detection.

**Table 2:** Associations between indoor pesticide concentrations and questionnaire responses by univariate linear regression analysis (*n* = 47).

Factor	*n*	Carbofuran	Chlorpyrifos	Fipronil	Cypermethrin	Tetramethrin
		β (%Change)	β (%Change)	β (%Change)	β (%Change)	β (%Change)
Cold season	21	−0.230 (−25.86)	−0.510 (−66.52)	−0.156 (−16.88)	−0.337 (−40.07)	−0.060 (−6.18)
Use of dehumidifiers	31	−0.184 (−20.2)	0.175 (19.12)	0.052 (5.33)	0.366 (44.19)	−0.227 (−25.48)
Use of air cleaners	9	−0.123 (−13.08)	0.211 (23.49)	0.023 (2.32)	0.276 (31.78)	−0.295 (−34.31)
Floor cleaning frequency						
Once a day	22	Referent	Referent	Referent	Referent	Referent
Once a week	19	−0.271 (−31.12)	−0.394 (−48.29)	−0.126 (−13.42)	−0.235 (−26.49)	0.025 (2.53)
Once a month	6	−0.058 (−5.97)	−0.551 (−73.49)	0.151 (16.29)	−0.682 (−97.78)	0.073 (7.57)
Detergents for floor cleaning	13	−0.295 (−34.31)	−0.010 (−1.00)	−0.536 (−70.90)	−0.309 (−36.20)	−0.531 (−70.06)
Age of house						
0–10 years	14	Referent	Referent	Referent	Referent	Referent
10–30 years	14	0.158 (17.11)	−0.423 (−52.65)	−0.703 (101.98)	−0.185 (−20.32)	0.398 (48.88)
>30 years	19	0.353 (42.33)	−0.073 (−7.57)	−0.489 (0.63)	−0.263 (30.08)	0.538 (71.25)
Frequent use of organic food	28	0.041 (4.18)	0.496 (64.21)	0.013 (1.30)	0.066 (6.82)	0.064 (6.60)
Nearby pesticide application	32	0.349 (41.76)	−0.146 (−15.71)	−0.287 (−33.24)	−0.068 (−7.03)	−0.292 (−33.91)
Indoor insecticide application	27	0.203 (22.5)	0.291 (33.77)	−0.306 (−35.79)	0.684 (98.17) *	0.364 (43.90)
Balcony or garden at home	34	−0.384 (−46.81)	−0.266 (−30.47)	0.123 (13.08)	−0.072 (−7.46)	−0.377 (−45.79)
Pets at home	23	−0.010 (−1.00)	−0.347 (−41.48)	0.747 (111.06) *	0.106 (11.18)	−0.026 (−2.63)
Tick treatment for pets	20	0.159 (17.23)	−0.121 (−12.86)	0.726 (106.67) *	0.342 (40.77)	0.102 (10.73)
Work involving pesticides	9	1.043 (183.77) **	0.813 (125.46)	0.554 (74.01) *	1.085 (195.94) *	1.294 (264.73) **
Cockroach observance (per week)						
None	24	Referent	Referent	Referent	Referent	Referent
Once or twice	14	−0.112 (−11.85)	0.193 (21.28)	0.565 (75.94)	0.256 (29.17)	−0.295 (−34.31)
3 times or above	9	−0.013 (−1.30)	−0.415 (−51.43)	0.603 (82.75)	0.466 (59.36)	0.507 (66.03)

*: significant at the 0.05 level (2-tailed); **: significant at the 0.01 level (2-tailed).

**Table 3:** Associations between indoor pesticide concentrations and questionnaire responses by multivariate linear regression analysis (*n* = 47).

Factor	*n*	Fipronil	Cypermethrin
		β (%Change)	β (%Change)
Indoor insecticide application	27	NA	−0.199 (−18.05)
Pets at home	23	0.568 (76.47)	NA
Tick treatment for pets	20	0.353 (186.62)	NA
Work involving pesticides	9	1.128 (208.9) **	1.053 (186.6) *

*: significant at the 0.05 level (2-tailed); **: significant at the 0.01 level (2-tailed); NA: not available.

**Table 4:** Coefficients of Spearman correlations between pesticide concentrations of indoor and outdoor dust.

Pesticides in Indoor Dust	Pesticides in Outdoor Dust					
	Carbofuran	Chlorpyrifos	Fipronil	Prallethrin	Cypermethrin	Tetramethrin
Carbofuran	0.407 *	0.549 **	0.340	0.410 *	0.470 **	0.575 **
Chlorpyrifos	0.567 **	0.840 **	0.464 **	0.488 **	0.390 *	0.543 **
Fipronil	0.368 *	0.096	0.769 **	0.201	0.108	0.222
Prallethrin	0.263	0.336	0.064	0.252	0.093	0.272
Cypermethrin	0.352 *	0.457 **	0.456 **	0.359 *	0.674 **	0.589 **
Tetramethrin	0.200	0.356 *	0.411 *	0.244	0.335	0.690 **

*: significant at the 0.05 level (2-tailed); **: significant at the 0.01 level (2-tailed).

**Table 5:** Spearman correlation between pesticides concentration in house dust and agriculture percent acreage within 50 m, 150 m, and 250 m buffer distance.

Type of Agricultural Area	BD	%Area (Mean ± SEM)	Carbofuran		Chlorpyrifos		Tetramethrin	
			Indoor	Outdoor	Indoor	Outdoor	Indoor	Outdoor
Non-specific agriculture land (0101)	50 m	24.77 ± 3.66	0.321 *	0.193	0.195	0.122	0.250	0.128
	150 m	32.16 ± 3.44	0.361 *	0.246	0.165	0.091	0.247	0.159
	250 m	37.43 ± 3.61	0.370 *	0.215	0.138	0.059	0.217	0.127
Rice cultivation area (010101)	50 m	6.74 ± 2.56	0.330 *	0.365 *	0.203	0.142	0.215	0.170
	150 m	9.27 ± 2.52	0.404 *	0.337 *	0.181	0.205	0.338 *	0.420 *
	250 m	11.63 ± 2.70	0.395 *	0.338 *	0.180	0.166	0.247	0.422 *
Crops‘ cultivation area (010102)	50 m	8.62 ± 2.41	0.140	0.265	0.231	0.085	0.006	0.072
	150 m	10.95 ± 1.61	0.093	0.201	0.021	−0.073	−0.005	−0.052
	250 m	12.59 ± 2.05	−0.014	0.185	0.023	−0.137	−0.117	−0.192
Fruits‘ cultivation area (010103)	50 m	6.33 ± 1.91	0.010	−0.105	−0.091	−0.114	0.114	0.078
	150 m	7.29 ± 1.41	0.044	−0.126	−0.079	−0.134	0.056	−0.016
	250 m	9.13 ± 1.40	0.122	−0.064	0.011	−0.072	0.073	−0.028
Abandoned cultivation area (010104)	50 m	3.09 ± 1.54	0.229	0.235	0.387 *	0.322 *	0.017	0.005
	150 m	3.47 ± 1.31	0.122	0.053	0.311 *	0.196	−0.061	−0.107
	250 m	3.65 ± 1.09	0.122	0.053	0.258	0.171	−0.178	−0.154

BD: buffer distance; SEM: standard error of mean; *: significant at the 0.05 level (2-tailed).

## Data Availability

The data presented in this study are available on request from the corresponding author. The data are not publicly available due to the ethical restriction.
